# Alzheimer’s Transgenic Model Is Characterized by Very Early Brain Network Alterations and β-CTF Fragment Accumulation: Reversal by β-Secretase Inhibition

**DOI:** 10.3389/fncel.2018.00121

**Published:** 2018-05-08

**Authors:** Siddhartha Mondragón-Rodríguez, Ning Gu, Frederic Manseau, Sylvain Williams

**Affiliations:** ^1^Department of Psychiatry, Douglas Mental Health University Institute, McGill University, Montreal, QC, Canada; ^2^CONACYT National Council for Science and Technology, Mexico City, Mexico; ^3^UNAM Developmental Neurobiology and Neurophysiology, Institute of Neurobiology, National Autonomous University of Mexico, Querétaro, Mexico; ^4^Department of Translational Neuroscience, The Royal Mental Health Research Institute, University of Ottawa, Ottawa, ON, Canada

**Keywords:** Alzheimer’s disease, hippocampus, CA1/subiculum area, amyloid-β, β-CTF

## Abstract

Alzheimer’s disease (AD) is defined by the presence of amyloid-β (Aβ) and tau protein aggregates. However, increasing data is suggesting that brain network alterations rather than protein deposition could account for the early pathogenesis of the disease. In the present study, we performed *in vitro* extracellular field recordings in the CA1/subiculum area of the hippocampus from 30 days old J20-TG-AD mice. Here, we found that theta oscillations were significantly less rhythmic than those recorded from control group. In addition, J20 mice displayed significantly less theta-gamma cross-frequency coupling (CFC) as peak modulation indexes for slow (25–45 Hz) and fast (150–250 Hz) gamma frequency oscillations were reduced. Because inhibitory parvalbumin (PV) cells play a vital role in coordinating hippocampal theta and gamma oscillations, whole-cell patch-clamp recordings and extracellular stimulation were performed to access their intrinsic and synaptic properties. Whereas neither the inhibitory output of local interneurons to pyramidal cells (PCs) (inhibitory→PC) nor the excitatory output of PCs to PV cells (PC→PV) differed between control and J20 animals, the intrinsic excitability of PV cells was reduced in J20 mice compared to controls. Interestingly, optogenetic activation of PV interneurons which can directly drive theta oscillations in the hippocampus, did not rescue CFC impairments, suggesting the latter did not simply result from alteration of the underlying theta rhythm. Altered young J20 mice was characterized by the presence of β-CTF, but not with Aβ accumulation, in the hippocampus. Importantly, the β secretase inhibitor AZD3839-AstraZeneca significantly rescued the abnormal early electrophysiological phenotype of J20 mice. In conclusion, our data show that brain network alterations precede the canonical Aβ protein deposition and that, such alterations can be related to β-CTF fragment.

## Introduction

Alzheimer’s disease (AD) is characterized by memory impairments that tend to correlate with abnormal protein aggregation in several areas of the brain, including the hippocampus (Gorevic et al., 1986; Garcia-Sierra et al., 2001; Mondragon-Rodriguez et al., 2014; Qian et al., 2017). Importantly, due to a coordinated synchronous activity of multiple neuronal assemblies, the hippocampus is known for being involved in memory storage and information processing (Moser et al., 2008; Kyle et al., 2015; Ryan et al., 2015; Bott et al., 2016). Although the exact mechanism behind this complex phenomenon remains unknown, rhythmic activity such as theta and gamma oscillations mainly generated by synchronous activity of multiple neuronal assemblies including parvalbumin positive (PV+) and pyramidal neurons, is thought to play an important role (Bott et al., 2016; Colgin, 2016; Fernandez-Ruiz et al., 2017). Furthermore, theta and gamma frequency interactions, a phenomenon named cross frequency coupling (CFC), has been proposed to be a critical component of memory processes (Belluscio et al., 2012; Chaieb et al., 2015; Bott et al., 2016). Not surprisingly, recent data showed that oscillatory activity in the theta and gamma frequency band is altered in AD patients that are characterized by protein deposition in several areas of the brain (van Deursen et al., 2008). Despite the degree of correlation between protein deposition and brain alterations, recent evidence has led the field to consider soluble Aβ peptides (e.g., dimers, trimers, tetramers, and higher oligomers) rather than insoluble aggregates, as the cause of synaptic dysfunction and network disorganization (Pena et al., 2006; Mondragon-Rodriguez et al., 2012; Mucke and Selkoe, 2012). In this regard, it is known that early amyloidogenic maturation of human amyloid β precursor protein (hAPP) involves processing by β-secretase which generates the intracellular β-C-terminal fragment (b-CTF) of hAPP (Lauritzen et al., 2016). Of importance, in the TgCRND8 mouse model of AD, our group reported early alterations of theta-gamma CFC in the principal output region of the hippocampus, the subiculum (Goutagny et al., 2013). Consistent with the above data, intracellular b-CTF fragments were found to be increased in the pyramidal cell (PC) layer of the CA1/CA2 hippocampal area (Mahar et al., 2016).

Aiming to explore if b-CTF could function as an important culprit in network alterations during very early AD development, we used a well-established *in vitro* septo-hippocampal preparation (Manseau et al., 2008; Goutagny et al., 2009) to study putative hippocampal network alterations in young (1-month-old) J20 mice. This transgenic mouse model of AD features high levels of Aβ overexpression, which result from the introduction human APP with two mutations (the Swedish and Indiana mutations i.e., APPSwe/Ind) linked to familial AD (Mucke et al., 2000). J20 mice develop plaques by the age of 5–7 months, exhibit altered electrophysiological network function, mostly reflected in hyperexcitability and epileptiform activity (Verret et al., 2012), and display deficits in learning and memory performance (Mably et al., 2015). At 1 month of age, however, there is no data that evaluate the network function. Remarkably, our results show that there is a progressive alteration in theta rhythm generation between the age of p15 to 1 month of age. Indeed, at this early developmental stage, a significant reduction in theta oscillations rhythmicity and in theta-gamma coupling was found. Importantly, young J20 mice was characterized by the presence of hippocampal b-CTF but not of Aβ. The use of the *in vitro* hippocampal preparation provided a unique opportunity for evaluating the potential mechanisms involved in network alterations. Surprisingly, optogenetic manipulation of theta power and frequency did not rescue the theta-gamma coupling impairments in the J20 model. Patch-clamp recordings, however, revealed a reduction in the excitability of subiculum PV+ interneurons which could account for instability of oscillatory activities. Finally, pharmacological blockade of b-CTF generation completely restored normal rhythmicity of theta oscillations in the J20 model.

## Materials and Methods

### Transgenic Mice

All animal handling procedures were in accordance with national and international guidelines and were approved by the ethical committee (McGill University). Mice expressing a mutant form of the human amyloid protein precursor bearing both the Swedish (K670N/M671L) and the Indiana (V717F) mutations (APP_SwInd_) under the control of the PDGFB promoter [B6. Cg-Tg(PDGFB-APP_SwInd_)] were obtained from The Jackson Laboratory.

### Hippocampal Preparation

Septo-hippocampal preparations were isolated from 30 to 39-day-old mice as previously described (Manseau et al., 2008; Goutagny et al., 2009; Manseau and Williams, 2017)., septo-hippocampal preparations were cut in sucrose-based ice-cold artificial cerebrospinal fluid (aCSF). In brief, one spatula supported the inner portion of the cortical hemisphere, and the other was used to gently pull away the brainstem and thalamus to expose the hippocampal artery and underlying CA3 and dentate gyrus (DG). To separate the hippocampus from the cortex the spatula was again placed between the cortex and extreme dorsal end of the hippocampus and moved smoothly through to the caudal portion of cortex. During this process the spatula helped separate the CA1/subicular tissue from the overlying cortex. The hippocampal complex was removed from the surrounding brain tissue by placing one spatula on the CA3/DG region of the dorsal hippocampus and pulling the dorsal hippocampus toward to the caudal portion of the brain. Blood vessels may impede prompt removal and should be cut away but not ripped out so as to avoid unnecessary tissue damage. The entire hippocampal isolation procedure was completed within one minute. Any remaining cortex was removed using micro-scissors when the preparation was returned to the oxygenated sucrose solution, then recovered in a submerged holding chamber containing aCSF at room temperature (22°C) for 40 min before use. ACSF contained (mM): 120 NaCl, 3 KCl, 2 MgSO4, 2.5 CaCl2, 1 NaH2PO4, 25 NaHCO3, 20 glucose, and was bubbled with 95%, O2 and 5% CO2.

### Electrophysiology and Field Recordings

Preparation was transferred to a submersion chamber and continuously perfused with ACSF at 30°C. Local field potentials (LFPs) were recorded using glass micropipettes (1–4 MΩ) filled with ACSF. Signals were recorded through a differential model 1700-AC amplifier (A–M Systems), filtered online (0.1 – 500 Hz), and sampled at 5 KHz. Signals were referenced to the bath medium and connected to ground.

The wave-shape of the theta oscillation varied according to frequency and maintained a symmetrical near-sinusoidal shape similar to *in vivo* theta rhythm. This hippocampal theta was observed using normal artificial cerebrospinal fluid. Power spectra were calculated using the multitaper method (Chronux toolbox) using seven tapers (Bokil et al., 2010). Integrated theta-band power was calculated in 5-s bins over a period of 2–14 min and the mean spectrum was taken as the grand mean of each animal. Changes in theta power were measured in mV^2^/Hz.

The modulation index (MI) was calculated using the algorithm described previously (Scheffer-Teixeira and Tort, 2016) and was used to test the phase locking of all frequencies in the range 5–400 Hz to the theta phase. We extracted the single filtered theta trace with bandpass filters set -1 to +1 Hz around the dominant theta frequency and calculated theta phase using the Hilbert transform. Phase was converted to degrees where -180° and +180° indicate the theta trough. The gamma amplitude was calculated by taking the filtered rectified gamma trace using the absolute value of the Hilbert-transformed gamma signal. The MI is normalized measure that reflects how well the instantaneous gamma amplitude is phase-locked to the underlying theta cycle. The MI was calculated between theta phase and each 5 Hz frequency band spanning 5–400 Hz. The MI value for each experiment was taken as the maximum MI value in the respective gamma band. Surrogate calculations were performed on the data by shifting the gamma amplitude signal by randomly selected increments and the original MI was required to >2SD above the mean surrogate MI to be considered statistically significant.

### Patch Recordings and Optogenetics

For optogenetic activation of PV cells, blue light (473 nm; intensity 35 mW) was delivered through a custom-made LED system (Luxeon) coupled to a 3-mm-wide fiber optic (Edmund Optics) and placed above the recorded area. Stimulus of sinusoidal light of constant amplitude and exponentially increasing frequency (0–12 Hz) was delivered to the cells (ZAP protocol). J20-PV-cre animals received intra-hippocampal injection of the Cre-dependant virus AAV-ChETA-eYFP at ∼15 days of age, rate 0.5–0.8 μl. Injection coordinates were set to obtain virus expression in both CA1 and SUB (anteroposterior 2.70 mm from bregma, lateral 3 mm, dorsoventral 2.05 mm). Pups were returned to their home cage and allowed to recover 2–3 weeks after surgery.

To assess electrophysiological properties of CA1/subiculum PV cells, whole-cell patch clamp recordings were performed in brain slices from J20-PV-cre animals which had received intra-hippocampal injection of the Cre-dependant virus AAVdj-EF1α-DIO-YFP at ∼15 days of age. Mice were euthanized at 30–39 days (15–23 days after viral injection), their brains were quickly extracted and placed in ice-cold oxygenated (95% O2/5% CO2 gas mixture) slicing solution containing the following (in mM): 252 sucrose, 26 NaHCO_3_, 10 glucose, 2.5 KCl, 4 MgCl_2_ 1.25 KH_2_PO_4_, 0.1 CaCl_2_ (pH 7.35). Coronal slices (300 μm) were cut using a vibratome (Leica VT1000S) and transferred to a holding chamber with oxygenated ACSF (room temperature) containing the following (in mM): 125 NaCl; 26 NaHCO_3_; 25 glucose; 2.5 KCl; 2 MgCl_2_; 1.25 NaH_2_PO_4_ and 2 CaCl_2_, pH 7.35. Slices were transferred to the recording chamber perfused with oxygenated (30°C) aCSF (2 ml/min). PV cells located within the CA1/subiculum region were visually identified by YFP fluorescence (488 nm, **Figure 3A**) using an upright Siskiyou microscope equipped with a 40x immersion objective (Olympus Canada, Richmond Hill, ON, Canada) and an excitation light source (X-cite Series 120Q, Lumen Dynamics, Mississauga, ON, Canada). Signals were amplified using a Multiclamp 700B patch-clamp amplifier, sampled at 20 kHz and filtered at 10 kHz using a Multiclamp 700B commander and analyzed using pClamp (Molecular Devices, Sunnyvale, CA, United States) as well as a custom written software (J. R. Huguenard^1^) for analyzing spontaneous events. Cell characterization was performed by injecting 0–600-pA, 600-ms-long, depolarizing and hyperpolarizing current steps. Patch electrodes (borosilicate glass, Warner Instruments, Hamden, CT, United States) had a resistance of 2–5 MΩ and were filled with either; (1) a standard intra-pipette solution containing (in mM) 144 potassium gluconate, 3 MgCl_2_, 10 HEPES, 0.2 EGTA, 2 ATP, and 0.3 GTP (pH 7.2, 290 mOsm) for excitatory post-synaptic current recordings, or (2) a “high-chloride” intra-pipette solution containing (in mM) 70 K- gluconate, 70 KCl, 2 NaCl, 2 MgCl_2_, 10 HEPES, 1 EGTA, 4 ATP and 0.3 GTP, and 5 QX-314 (pH 7.3, 290 mOsm) for inhibitory post-synaptic current recordings (Manseau et al., 2010). Based on the Nernst equation, the estimated *E*_Cl_ of the “high-chloride” solution was -16 mV, without correction for liquid junction potential. Action potentials were blocked with QX-314 (5 mM). Under these conditions, monosynaptically elicited IPSCs recorded in PCs with *V*_m_ clamped at -70 mV were represented by large inward currents (**Figure 3G**). Extracellular stimulation was performed using single-current pulses (0.05–0.9 ms, 10–100 μA) delivered every 3 s with a A360 stimulus isolator (WPI) through a glass pipette monopolar electrode (filled with ACSF and used as a holder for the connected silver wire). For testing CA1 input to subiculum PV cells, a stimulation electrode was placed in or near the PC layer or deep oriens where fibers project between the two areas (O’Mara, 2005). For testing local inhibition from subiculum PV cells, eIPSCs were evoked monosynaptically by placing the stimulating pipette ∼50 μm away from the recorded pyramidal cells (PCs). DNQX [10 μM] and APV [25 μM] were added to block fast glutamatergic transmission and isolate GABAergic currents during recordings from subiculum PCs. Single-pulse electrical stimuli (50 μs pulses; 0.3 Hz; intensity range 10–100 μA) were used to find the stimulation intensity for threshold responses (∼50% failure rate), this intensity was then applied while incrementing pulse duration (range 50–900 μs) to elicit post-synaptic currents that were used to build input/output (I/O) curves for each neuron. The membrane potential was clamped at -70 for eEPSC and eIPSC recordings. Recordings were restricted to neurons located within the CA1/subiculum portion of the hippocampus (between the proximal and middle-third portion of the subiculum). Whole-cell recordings were kept for analysis only if the neuron remained stationary throughout the recording, spikes overshot 0 mV, and access resistance was <30 MΩ.

### Immunofluorescence

For label immunofluorescence, sections were blocked with 10% NGS (Sigma) in PBS for 30 min. Double labeling experiments were conducted by combining two of the primary antibodies. The following antibodies and dilutions were used: (PV m-Ab against PV, Invitrogen, 1:2000) and (CT20 Rb-Ab against β-CTF, Millipore, 1:1000). Bound primary mouse and rabbit antibodies were detected with Alexa Fluor Secondary Antibodies 488 and 568 (Thermo-Fisher Scientific, 1:4000). In all experiments (*n* = 8, non-TG and 7, TG), incubation with primary antibodies was overnight at 4°C, followed by 2 h at room temperature with corresponding secondary antibodies. The sections were mounted in antiquenching medium (Vectashield, Vector Laboratories, Inc., Burlingame, CA, United States). Labeled brain sections were viewed with a 20X and 5X Plan-Apochromat on a Carl Zeiss Microscope (Germany).

### AZD3839 Assay

The mice (*n* = 5–6) received vehicle or AZD3839 at 200 μmol/kg as a single dose or 100 μmol/kg as repeated doses twice daily for 7 days via intraperitoneal injection (IP). Drug were store in dry, dark and -20°C.

### ELISA Experiments

β-CTF fragment content in one-month-old J20 hippocampal samples were quantified with sandwich ELISA kit (Immuno-Biological Laboratories Co., Ltd. Japan). To identify transgenic animals, standard PCR and gel electrophoresis on a 1.5% agarose gel was performed (according to The Jackson Laboratory genotyping protocols, United States).

### Data Analysis-Using Custom MATLAB Software

Field traces were filtered and analyzed using a Fourier transform. Autocorrelogram of the filtered theta signal was used to derive a value reflecting the degree of rhythmicity. Unless otherwise stated, all data were analyzed statistically by either, pairwise comparisons with Student’s *t*-tests or Mann–Whitney *U*-test using STATISTICA 7 (StatSoft, Inc.). In all tests, values of *p < 0.05* were considered to indicate significance. Bar graphs show experimental mean, with error bars indicating standard error of the mean.

## Results

### Network Oscillatory Activity at Theta Band Frequency Is Altered in Young J20 Mice

**FIGURE 1:**
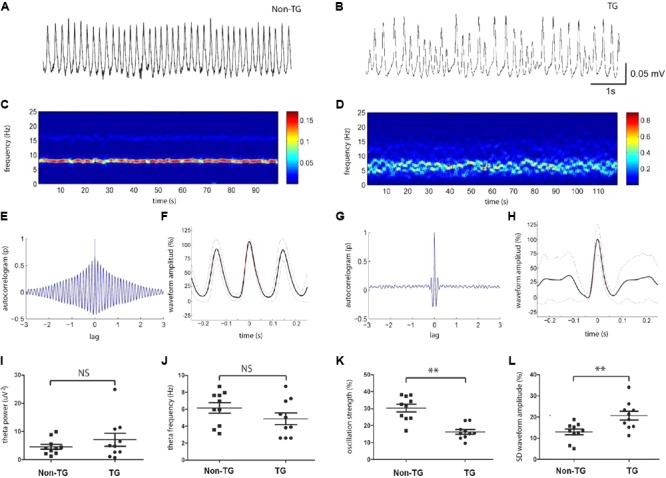
Hippocampal theta activity was disrupted in young J20 mice. Representative trace of theta oscillation recorded at hippocampal ca1/subiculum area from J20 transgenic (TG) mice at age of P30 **(B)**, in comparison to the trace recorded from Non-transgenic (Non-TG) animal **(A)**. Spectrogram (100 s) from the same recordings shown above **(C,D)**. Autocorrelation of theta oscillations from TG and Non-TG mice **(E,G)**. Averaged oscillation waveform from 100 s recordings with standard deviation (**F,H**, red dashed line). Statistics showed the oscillation power **(I)** and frequency **(J)** had no significant difference between TG and Non-TG group. **(K)** The oscillation strength from J20 group are significantly lower than those from the control group. **(L)** The standard deviation of the averaged waveform peak is significantly higher in J20 group compares to Non-TG group (*n* = 10 in each group, ^∗∗^*p* < 0.01 and NS *p* < 0.05).

As previously reported, network oscillatory activity can be measured via extracellular field recordings in the complete septo-hippocampal preparation *in vitro* (Manseau et al., 2008; Goutagny et al., 2009). Using this preparation, we examined the properties and possible alterations of endogenous network oscillations in the CA1/subiculum area of J20 transgenic mice (TG) compared to family related non-transgenic (Non-TG) mice. Importantly, all recordings were performed at 30 days post-natal (p30–39 age) in both groups (*n* = 10, non-TG-p34.6 and 10, TG-p35.2). In agreement with previous studies done in the CRND8 mouse AD model (Goutagny et al., 2009; Jackson et al., 2011), hippocampi from control J20 animals showed spontaneous and robust rhythmic theta frequency (3–8 Hz) oscillations in CA1 and subiculum areas (**Figure 1A**). In contrast to our previous published results, oscillations recorded from the J20 mice displayed considerably reduced rhythmicity (**Figure 1B**). As shown with spectrogram analysis (**Figure 1C**), theta oscillation rhythmicity was more regular in Non-TG animals when compared to the TG group (**Figure 1D**). Autocorrelations and averaged waveform further confirmed the reduced rhythmicity in TG mice (**Figures 1E,F**) when compared to Non-TG mice (**Figures 1G,H**). The average frequency (4.88 ± 0.69 Hz; *n* = 10) and power (7.07 ± 2.2 μV^2^, *n* = 10) of the theta oscillations from TG group were similar to those recorded from Non-TG group (frequency, 6.15 ± 0.62 Hz; power 4.58 ± 0.89 μV^2^, *n* = 10, two tail *t-*test: *p* > 0.05) (**Figures 1I,J**). The average oscillation strengths (index of rhythmicity, see Materials and Methods) of theta oscillations recorded in Non-TG group (30.3 ± 2.2%, *n* = 10) were significantly higher than those recorded from the TG group (16.3 ± 1.3%, *n = 10, p* < 0.01, **Figure 1K**). The standard deviation of the waveform amplitudes recorded from Non-TG group (13 ± 1, *n* = 10) were significantly inferior to those recorded from TG group (21 ± 2, *n* = 10, *p* < 0.01, **Figure 1L**). In summary, within the theta frequency band, our analysis revealed network alterations in young TG animals when compared to control Non-TG animals. Those alterations were reflected in the reduction of oscillation strength (rhythmicity values) and increased SD of waveform amplitudes, but not in either, total power or frequency of theta oscillations.

### Theta-Gamma Cross-Frequency Coupling Is Reduced in J20 Mice

Our group recently reported the presence of slow (SG) and fast gamma (FG) oscillatory activity nested in theta oscillations in the whole isolated hippocampus preparation (Jackson et al., 2011). As previously mentioned, our laboratory also reported that in the TgCRND8-AD mouse model, robust alterations of theta-gamma CFC were detected in the hippocampal subiculum before Aβ accumulation (Goutagny et al., 2013).

**FIGURE 2:**
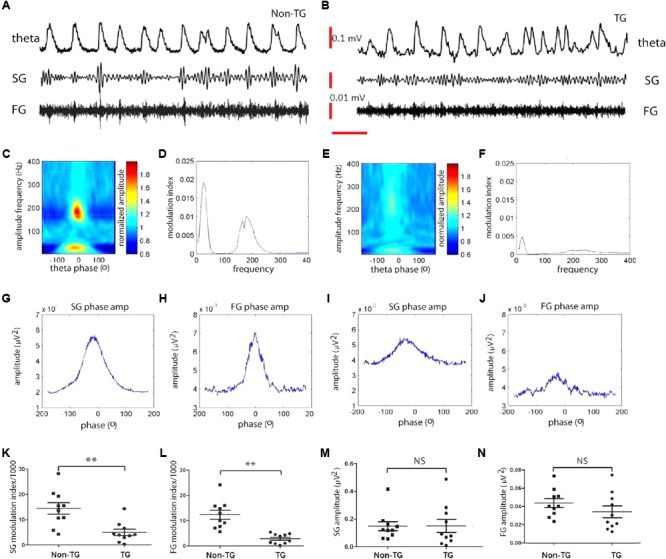
Theta-gamma cross frequency coupling (CFC) is affected as early as 1-month in J20 model. Representative trace filtered at theta (2–12Hz), slow gamma (25–55Hz), and fast gamma (150–250Hz) frequency band recorded from non-transgenic (Non-TG) and transgenic (TG) group **(A,B)**. CFC between theta phase and gamma amplitude **(C,E)**. Red plot indicates strong coupling to theta phase **(C,E)**. Right plot, the modulation index (MI) value was measured to reflect how well the gamma amplitude is phase locked to the on-going theta cycles **(D,F)**. Gamma amplitude modulation by theta phase in Non-TG and TG, respectively, slow gamma **(G,I)** and high gamma **(H,J)**. The MI value of slow gamma and fast gamma are significantly lower in TG group than those from Non-TG group, as shown in **K** and **L**. The reduction of MI value was not due to gamma power change as shown in **M** and **N**. The TG and Non-TG group have similar slow gamma and fast gamma power **(M,N)**. (*n* = 10 in each group, *t*-test, ^∗∗^: *p* < 0.01, NS: *p* > 0.05.

To investigate whether theta and gamma oscillatory activity properties were similarly altered in the J20 mice, we assessed theta-gamma CFC in the hippocampal subiculum area of p30 mice and compared it to that of Non-TG mice. Surprisingly, we found that in both groups, theta and gamma rhythms could be recorded with similar power (**Figures 2A,B**). Furthermore, within the slow (25–45 Hz) and fast (150–250 Hz) gamma range, we found strong CFC to the theta phase in Non-TG mice (**Figures 2C,D**). We used the MI based on a quantification method recently developed by Tort et al., (Tort et al., 2008) which reflects how phase-locked the instantaneous gamma amplitude is to the underlying theta cycle (**Figure 2D**). In contrast to the control group, TG mice showed reduced CFC (**Figure 2E**) and low MI (**Figure 2F**). In control mice, both SG and FG amplitudes were significantly modulated by theta phase (**Figures 2G,H**). However, the gamma amplitude modulation was severely disrupted in TG mice (**Figures 2I,J**). Summary data further confirmed that TG mice display a significant decrease in cross-frequency theta-SG coupling compared to controls animals (MI × 10^-3^: 4.96 ± 1.26 in TG vs. 14.39 ± 2.25 in Non-TG; *t*(18) = 3.66, *p* = 0.0018; **Figure 2K**), and significant decrease in Theta-FG CFC (MI × 10^-3^: 2.84 ± 0.61 in TG vs. 12.29 ± 1.76 in Non-Tg; *t*(18) = 5.067, *p* < 0.001; **Figure 2L**). The power of the SG recorded in TG mice showed no significant change when compared to control group (SG power: 0.15 ± 0.04 μV^2^ in J20 vs. 0.15 ± 0.03 μV^2^ in controls; *t*(18) = 0.047, *p* = 0.96; **Figure 2M**); FG power was also found to be similar in both groups (FG power: 0.034 ± 0.006 *N* = 10 μV^2^ in J20 vs. 0.043 ± 0.005 μV^2^ in controls; *t*(18) = 1.192, *p* = 0.25; **Figure 2N**), suggesting that the reduction of MI values was not simply due to a change in gamma power. Taken together, these data indicate that young J20 mice exhibit severe disruption in theta/gamma CFC, in both theta/slow gamma and theta/fast gamma.

### Excitability of PV Interneurons Is Reduced in J20 Mice

**FIGURE 3:**
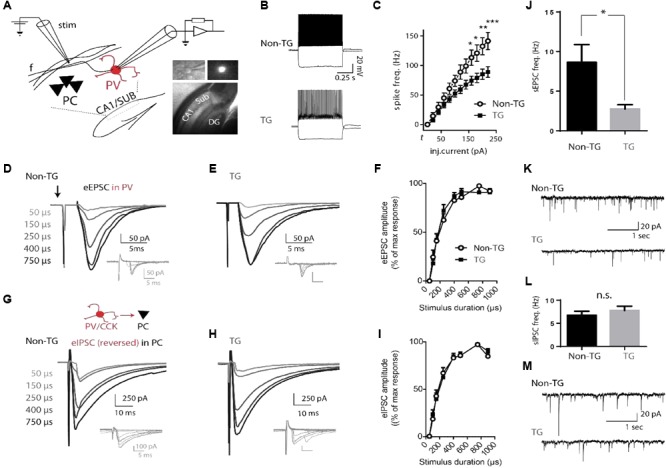
Altered physiological properties of hippocampal PV interneurons in J20 mice. **(A)** Schematic drawing of the CA1/SUB region of a hippocampal slice showing the location of a recorded PV cell relative to a stimulation (Stim) electrode. Insert: representative images of a hippocampal slice and YFP-expressing fluorescent PV cell recorded in patch (40X). PC, pyramidal cell layer; f, CA1-subiculum fibers; DG, dentate gyrus. **(B)** Fast-spiking (FS) firing-pattern evoked in PV neurons from Non-TG and TG J20-PV-Cre animals (top and bottom, respectively) in response to depolarizing current steps. Hyperpolarizing steps essentially show absence of voltage-dependent inward rectification (depolarizing sag) and associated rebound firing. **(C)** Plot of mean firing frequencies in response to injected currents of various supra-threshold amplitudes in PV interneurons from Non-TG (open circles) and TG mice (solid squares). Current injections were 600 ms square pulses. Firing-frequencies are plotted from threshold current (*t*) to *t* + 200 pA. Note that PV cells from both groups show typical fast-spiking (FS) properties but that a significant group-specific difference in firing frequency is evident at current injection levels shown. **(D,E)** Representative examples of CA1 evoked excitatory post-synaptic currents (eEPSCs) recorded in PV cells from non-TG and TG mice. Each trace (average of 5–10 trials) was evoked by increasing the duration of the minimal current pulse, from threshold to maximum (50–900 μs), without changing the stimulus amplitude. Insets (gray traces below) show successive responses to minimal stimulation (with 5/10 failures in **D** and 4/10 failures in **E**). Extracellular electrical stimuli were delivered at low-frequency (0.33 Hz) through a glass pipette placed 100–200 μm from the soma of the recorded PV cell (in the stratum pyramidale/oriens, toward the CA1/sub border). Stimulus artifact is indicated by the arrow pointing down. Evoked EPSCs increased monotonically with stimulus duration until saturation at ∼400 μs. **(F)** Plot of mean eEPSC amplitude in response to stimuli of increasing duration for PV cells from Non-TG versus TG groups. The EPSC evoked at each stimulus duration was normalized to the maximal response of each neuron. Overall a two-way ANOVA indicated that the amplitude of CA1-projection evoked excitatory post-synaptic currents (eEPSCs) did not differ between Non-TG and TG mice. **G** and **H**, evoked inhibitory post-synaptic currents (eIPSCs) recorded in pyramidal neurons (average of 10 trials per trace) during activation of local inhibitory cells at various stimulation intensities in non-TG and TG mice. Insets (gray traces) show responses to minimal stimulation (4/10 failures in **G**; 6/10 failures in **H**). **(I)** Mean normalized amplitude of eIPSCs elicited in pyramidal neurons by electrical stimuli in local GABAergic cells. **(J,K)** mean frequency of spontaneous EPSCs (bar graph) recorded from PV cells in non-TG and TG mice and representative example. **(L,M)** same as **J** and **K** for spontaneous IPSCs recorded from PCs in non-TG and TG mice (*n* = 10, non-TG and 8, TG).

To identify a potential cellular mechanism for neuronal network alterations in young J20 mice, we next performed whole-cell patch-clamp recordings in acute hippocampal slices. As parvalbumin (PV)-containing GABAergic interneurons target the perisomatic region of PCs, they have long been considered to exert powerful control over the output of large populations of principal cells (Cobb et al., 1995) and play a critical role in synchronizing rhythmic activity of the hippocampus (Buzsaki and Eidelberg, 1983; Klausberger et al., 2003; Amilhon et al., 2015). We therefore focused on characterizing the basic electrophysiological properties of PV cells in the CA1/subiculum area. Mice from a double-transgenic J20-PV-Cre colony (non-TG and TG, see section “Materials and Methods”) received intra-hippocampal injections of a Cre-dependent adeno-associated virus (AAVdj-EF1α-DIO-YFP) allowing expression of the yellow fluorescent protein (YFP) in PV neurons (see **Figure 3A** and section “Materials and Methods”). PV cells distributed along the CA1/subiculum principal cell layer, were identified by the presence of YFP fluorescence and responded to depolarizing current steps with fast, non-adapting, high-frequency firing behavior (**Figures 3A,B**). These cells did not respond to hyperpolarizing pulses with rebound bursts of action potentials. The mean values for their resting potential, membrane resistance, spike amplitude, action potential threshold, spike half-width and AHP amplitude showed no statistically significant differences between non-TG and TG mice (**Table 1**).

**Table 1:** Membrane and firing properties of PV interneurons in J20-PV-Cre mice.

	Non-transgenic	Transgenic
Resting potential (mV)	–62.3 ± 3.31	–57.5 ± 4.39
Membrane resistance (MΩ)	190.4 ± 42.4	199.8 ± 41.9
Spike amplitude (mV)	83.4 ± 3.47	82.92 ± 2.61
Spike half width (ms)	0.52 ± 0.042	0.59 ± 0.05
^a^ Sag amplitude (mV)	1.36 ± 0.12	1.71 ± 0.45
^b^ AHP amplitude (mV)	–17.02 ± 1.53	–17.29 ± 1.59
Action potential threshold (mV)	–39.2 ± 1.92	–42.3 ± 1.65
^c^ Maximal firing frequency (Hz)	141.5 ± 14.57	89 ± 9.05^∗∗^
^d^ Spike frequency adaptation (%)	14.24 ± 5.83	34.42 ± 6.51^∗^
^e^ f–i slope (Hz/pA)	0.56 ± 0.07	0.31 ± 0.03^∗∗^

*All values are means ± SEM; *n* = 10 for PV interneurons from non-transgenic mice; *n* = 10 for PV interneurons from transgenic mice. Independent *t*-test comparisons produced significant differences for maximal firing frequency (*P* < 0.005), spike frequency adaptation (*P* < 0.05), and f–*i* slope (*P* < 0.005). ^*a*^The presence of a hyperpolarization-activated current (Ih)-dependant voltage-dependent inward rectification (depolarizing “sag”) was examined in response to hyperpolarizing current pulses and its amplitude was measured for current pulses inducing an initial hyperpolarization to -100 mV. ^*b*^Afterhyperpolarization (AHP) was calculated as the difference between of the first isolated action potential threshold and the most negative level reached during the repolarizing phase. ^*c*^Maximal firing frequency was calculated from the total number of spikes elicited by 600-msec-long current injections of 200 pA above the firing threshold (*t*). ^*d*^Spike frequency adaptation was calculated as the normalized ratio of the instantaneous frequency at the first and last spike intervals in action potential trains (1rst-last/1rst) at *t* + 200 pA. ^*e*^f–*i* slope, firing frequency versus injected current slope, with current injections ranging from 100 to 200 pA above threshold.*

To further characterize the intrinsic properties of PV neurons, an input-output (I/O) plot of the number of evoked action potentials as a function of injected current was generated (**Figure 3C**). Qualitatively, the firing pattern of PV neurons from both non-TG and TG mice displayed a nearly linear relationship throughout the stimulus intensity range (from threshold-current step (*t*) to *t* +200 μA). In these neurons, the firing rate increased progressively with stimulation amplitude with little or no evidence of time-dependent decrease in action potential discharge or spike-frequency adaptation (**Table 1** and **Figures 3B,C**). However, the maximum firing rate, as well as the slope of the firing response (firing frequency versus injected current), were both significantly reduced in PV cells from TG animals when compared to PV cells from Non-TG mice (*p* < 0.005 in both cases). Conversely, spike-frequency adaptation was found to be increased in TG mice (*p* < 0.05). Overall, a two-way ANOVA indicated that the I/O function was significantly different between PV neurons from non-TG and TG mice and that PV neurons from non-TG mice responded to current stimulation by generating a larger number of action potentials than those of TG mice. Indeed, a significant interaction existed (*p* < 0.0001), where depolarizing currents led to significantly higher firing rates for PV neurons from control mice than in TG mice (Bonferroni post-tests: *p* < 0.05 at 140–160 pA above threshold, *p* < 0.01 at 180 pA and *p* < 0.001 at 200 pA above threshold).

### Monosynaptic Excitation From CA1 to Subiculum PV Cells Is Unaltered in J20 Animals

Since reduced excitability of subiculum PV interneurons could reflect a change of their functional integration into hippocampal circuitry, we next examined the post-synaptic response of these cells to excitatory CA1 input. YFP-expressing PV cells were recorded in voltage-clamp (vc) mode (*V*_h_ = -70 mV) and tested during electrical stimulation of CA1 projections (see section “Materials and Methods”). In agreement with results from previous reports (Behr et al., 1998; Fricker and Miles, 2000; Knopp et al., 2005), stimulation of the CA1 efferent pathway elicited post-synaptic responses in both PCs (data not shown) and interneurons of the subiculum (**Figures 3D–F**). In PV cells from control and TG animals (*n* = 10 cells, recorded from 6 non-TG mice; *n* = 8 cells, recorded from 5 TG mice), minimal stimulation (50 μs pulses; 0.3 Hz; threshold intensity = 10–100 μA) evoked excitatory post-synaptic currents (eEPSCs) of relatively large amplitude (-49.02 ± 9.46 and -34.97 ± 5.89 pA; non-TG vs. TG, ns, **Figures 3D,E** insets) with peak current fluctuations (coefficient of variation, 50.29 ± 7.3, *n* = 9; 48.38 ± 5.18, *n* = 8, ns), brief half-width (1.51 ± 0.26 and 2.83 ± 0.82 ms; ns), fast rise-time (10%–90% rise times: 0.62 ± 0.1 and 1.25 ± 0.29 ms for non-TG vs. TG; *p* = 0.017), fixed latencies (mean latency measured from the onset of the stimulus current: 4.7 ± 0.88 and 6.34 ± 1.53 ms, ns), and frequent transmission failures (the criteria for threshold stimulation intensity was ∼50% failure rate).

The mean amplitude of eEPSCs recorded from PV interneurons increased when stimulus duration was raised above threshold and displayed a clear saturation (at approximately 400 μs) when stimulus duration was increased further (range 50–900 μs). The plot of eEPSC amplitudes versus stimulus duration displayed a roughly parabolic relationship in both control and TG mice; eEPSCs increased progressively at low stimulation intensity, reached a peak at approximately 400 μs (∼45% of maximum stimulation), and remained at a steady level thereafter (**Figures 3D–F**). No statistical difference was found in the input/output relationship of eEPSCs recorded in PV cells from control and J20-TG mice (two-way ANOVA). Interestingly, consistent with a recent report (Verret et al., 2012), the frequency of spontaneous EPSCs was higher in PV cells from controls than from J20-TG mice (**Figure 3J,K**). No differences were found in the amplitude of Type 1 events recorded for the two populations (Non-TG: -19.56 ± 1.1 mV, *n* = 10 vs. TG: -19.35 ± 1.6, *n* = 8). These results nevertheless suggest that a change in the intrinsic or network properties of principal cells could exist in J20 mice.

### The Synaptic Output of CA1/SUB Local Interneurons Is Unaltered in J20 Mice

While directly assessing the CA1 excitatory input to subiculum PV cells did not reveal any alteration in J20 mice, a disturbance in the inhibitory control of subicular burst-spiking PCs provided by local interneurons (Menendez et al., 2003), could lead to a severe disruption in hippocampal oscillations. To test this hypothesis, we used whole-cell recordings and local stimulation to examine the functional output of local inhibitory neurons onto CA1/Sub PCs in J20 mice. Monosynaptic IPSCs evoked in CA1/SUB pyramidal neurons were pharmacologically isolated in the continuous presence of ionotropic glutamate receptor antagonists DNQX [10 μM] and APV [25 μM]. IPSCs evoked at threshold intensity (**Figures 3G,H** insets) showed large peak current fluctuations (peak amplitude -187.4 ± 57.87 pA and -117.1 ± 36.46 pA; coefficient of variation (CV) 0.56 ± 0.05 and 0.65 ± 0.1 in non-TG and TG, respectively; *n* = 16 and 14), as well as brief and fixed latency (6.41 ± 0.58 and 7.5 ± 0.68 ms), occasional transmission failures, and fast rise times (mean rise time, 1.9 ± 0.4 and 2.5 ± 0.4 ms; all values for non-TG (*n* = 16) and TG (*n* = 14) respectively). No statistical difference was found in values reported above.

Pyramidal cells from Non-TG and transgenic PV-Cre J20 mice showed clear GABAergic responses to local stimulation and both groups displayed similar input-output curves as they reached a maximum response at the same stimulation intensity (**Figures 3G–I**, no significant difference, two-way ANOVA). The properties of spontaneous IPSCs (sIPSC) also did not differ between Non-TG and TG groups (frequency 6.8 ± 0.8 vs. 7.8 ± 0.9, peak amplitude -27.9 ± 4.5 vs. -26.6 ± 2.5, rise-time 0.9 ± 0.05 vs. 0.9 ± 0.05, half width 9.2 ± 0.5 vs. 8.9 ± 0.9) (**Figures 3L,M**). These findings suggest that the properties of GABAergic synapses from CA1/sub interneurons are not altered in J20 mice.

### Assessment of Extracellular Aβ and Intracellular b-CTF in J20 Mice

**FIGURE 4:**
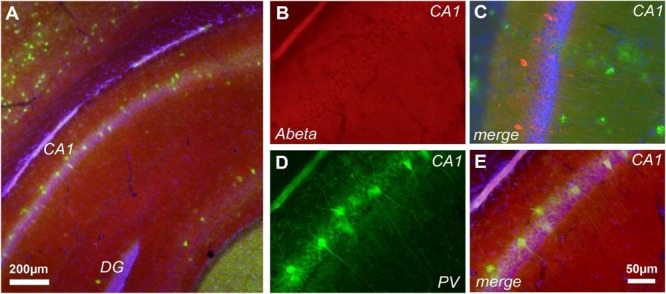
Aβ is not detected at 1-month of age in J20 mice. Immunohistochemical staining in the hippocampus of J20 mice with FCA3340 (red), PV (green) and DAPI (blue) showed no traces of Aβ in either areas of hippocampal formation **(A,B,D)**. Accordingly, no traces of Aβ were detected coexisting with the PV+ cells **(D,E)**. FCA3340 (green) reveals the presence of Aβ around CA1 in 5-month-old TG mice **(C)**, *n* = 3, non-TG and 4, TG.

Previous studies demonstrated that cognitive and brain network alterations in J20 AD model were correlated with Aβ deposition (Harris et al., 2010; Verret et al., 2012). However, assessment of Aβ with the FCA3340 antibody, which specifically recognizes human Aβ but not APP (Barelli et al., 1997), demonstrated no reactivity in hippocampus from 30 days old J20 mice (**Figures 4A,B**). Accordingly, no reactivity was observed coexisting with the PV+ cells (**Figures 4D,E**). FCA3340 (green) reveals the presence of Aβ around CA1 in 5-month-old TG mice (C).

**FIGURE 5:**
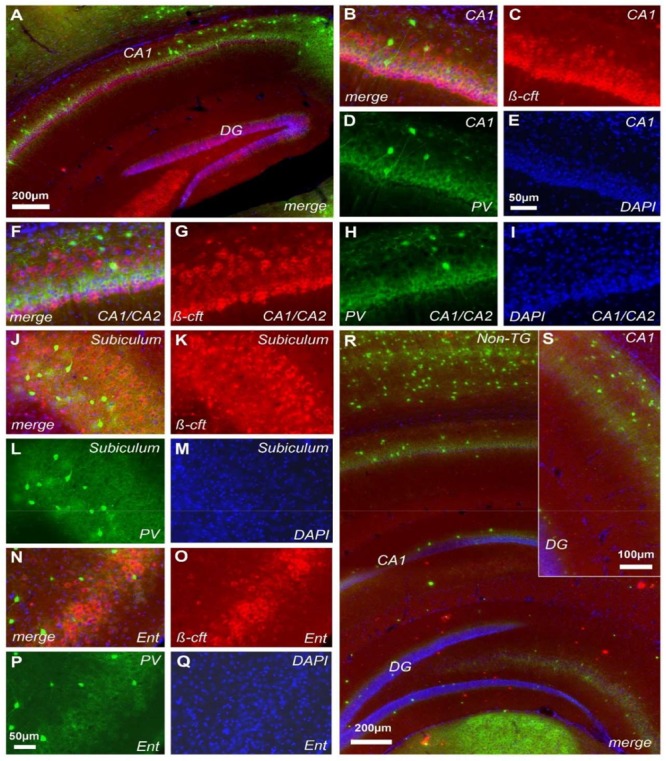
Cerebral b-CTF deposition in J20 mice. Immunohistochemical staining in the hippocampus of J20-TG mice with CT20 (red), PV (green), and DAPI (blue) reveals the presence of b-CTF in CA1 and DG **(A)**. Further characterization showed that b-CTF is predominantly present in the deeper pyramidal layer along CA1 **(B–I)**. b-CTF was also found in the subiculum **(J–M)** and entorhinal cortex **(N–Q)**. Control mice did not display b-CTF presence in the hippocampal formation **(R,S)**. See magnification in S7, *n* = 8, non-TG and 7, TG.

Of note, the CT20 antibody that recognizes the C-terminal intracellular fragment (b-CTF), revealed positive pyramidal hippocampal neurons (**Figure 5**). At p30 we observed a clear increase in the DG as revealed by CT20 labeling (**Figure 5A**, red color). The expression was clearly observed in the CA1 formation (**Figures 5A–C**). Notably, the increased expression was mainly around the deep pyramidal layer from CA1 (**Figures 5B,C**). Following the same pattern, the CA1/CA2 transition zone showed a similar distribution for b-CTF (**Figures 5F–I**). Again, most b-CTF increase was observed along the deep pyramidal layer (**Figures 5F–I**). The subiculum and entorhinal regions were also characterized by b-CTF increase (**Figures 5J–Q**). Non-TG mice did not show any detectable traces of b-CTF at any brain region of interest (**Figures 5R,S**). CT20 antibody showed no reactivity in Non-TG sample (**Figure 5S**). To evaluate whether the neurons that exhibited increased b-CTF levels were PV positive interneurons, we performed double-labeling for b-CTF (**Figure 5**, red color) and PV (**Figure 5**, green color). Imaging study revealed that positive PV cells barely express detectable levels of b-CTF (**Figures 5B,F,J,N**). Indeed, the intense PV labeling was observed between the deep pyramidal layer and the superficial pyramidal layer (**Figure 5F**).

### At Initial Stages, the Hippocampal Accumulation of b-CTF Becomes a Signature in J20 Mice

**FIGURE 6:**
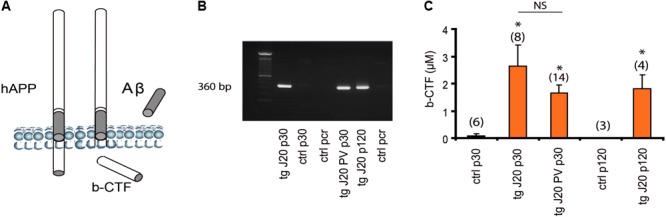
Accumulation of b-CTF in J20 mice. hAPP processing results in intracellular fragment b-CTF, the Aβ peptide and secondary products **(A)**. Detection of hAPP gene by gel electrophoresis on a 1.5% agarose gel. 50- to 1,000-bp size markers; lane 1, J20-TG positive; lane 2, J20 Non-TG; lane 3, pcr ctl; lane 4 J20-PV-Cre and lane 5, J20-TG p120 **(B)**. Levels of b-CTF at 30 and 120 days **(C)**. Data are expressed as mean (± standard error of the mean) percentage of media control b b-CTF levels. **(C)** Both p30 J20-TG, and p30 J20-PV showed significant levels of b-CTF as detected in a sandwich ELISA (IBL, hAPP b-CTF Assay Kit, Japan). At p120 J20-TG mice showed more that 50% increase in b-CTF **(C)**. Neither of the control had any significant b-CTF levels **(C)**, *p* < 0.005, data are expressed as mean (± standard error of the mean) percentage of media control b-CTF levels. ^∗^*p* < 0.005 and NS *p* > 0.05.

The intracellular b-CTF fragment is produced by the activity of β-secretase (BACE1) and has been reported to be present as early as 30 days of age in TGCRND8-AD model (Cavanagh et al., 2013). Our animals were carriers for the transgene (360 bp, **Figures 6A,B**) that expresses the mutant human amyloid protein precursor (hAPP) as previously mentioned (see section “Materials and Methods”). Like the TGCRND8 mice, the J20 mice were also characterized by significant increase levels in b-CTF fragment (**Figure 6C**, *p* < 0.005). The b-CTF levels were detected specifically in the hippocampus of TG mice (**Figure 6**). Again, and similarly to the TGCRND8 mice, our results revealed a significant increase in b-CTF levels (*p* < 0.005) at 30 days of age when compared to Non-TG mice (**Figure 6C**). The b-CTF remained significantly increased until up to 120 days of age (**Figure 6C**). Knowing that hippocampal oscillatory rhythms are supported by inhibitory parvalbumin (PV) cells (Amilhon et al., 2015), we developed a double TG mouse line that carries the 360 bp gene and is also PV-Cre dependent (J20-PV-Cre). Importantly, J20-PV-Cre mice displayed the same phenotype as the original J20 mice. Like the J20 mice, at 30 days of age, the J20-PV-Cre mice was also comprised with significantly increased levels in b-CTF compared to controls (**Figure 6C**, *p* < 0.005). Again, J20-PV-Cre Non-TG mice did not show any detectable levels in b-CTF at any age (data not shown).

### Optogenetic Investigation of Theta-Gamma Coupling Properties in J20 Mice

**FIGURE 7:**
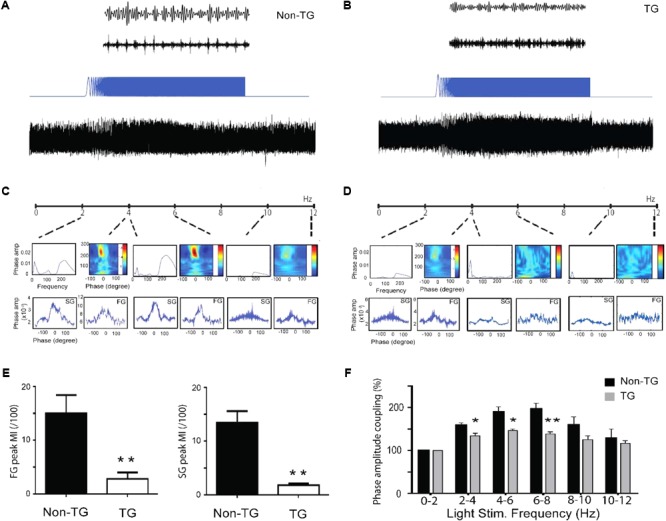
Altered oscillatory activity properties under optogenetic activation of PV neurons in hippocampus networks. Sample traces from Non-TG mice **(A)** and TG mice **(B)** showing 8 Hz (theta) light stimulation of hippocampal PV neurons with Cheta virus expression. Theta traces was then filtered at the 35–45 Hz slow gamma and 180–200 Hz fast gamma range. By using linear increase of the stimulation frequency from 0 to 12 Hz (ZAP protocol), both Non-TG and TG network oscillation can be evoked from 0 to 12 Hz **(A,B)**. Theta-gamma coupling properties are evaluated at different frequency range from both groups **(C,D)**. Summary graph showing the modulation index of FG and SG from TG are significantly lower than the Non-TG group **(E)**, (*t*-test, *n* = 5 in each group, *p* < 0.05). The TG group displayed reduced theta power at frequency range of 2–4 Hz, 4–6 Hz, and 6–8 Hz, compare to Non-TG group **(F)**, two tailed *t*-test, *n* = 5 in each group, ^∗^*p* < 0.05, ^∗∗^*p* < 0.01.

The observation of reduced theta-gamma coupling in J20 mice raises the possibility that the reduced coupling is a consequence of altered theta-strength (rhythmicity). To optimally control for theta oscillation power and frequency differences between the TG and Non-TG groups, we used optogenetics to activate PV neurons in the hippocampal subiculum region (Amilhon et al., 2015). By using linearly increased sinusoidal frequency (ZAP protocol) from 0 to 12 Hz, both Non-TG mice and TG mice were reliably driven by light stimulation eliciting theta oscillation, as well as gamma rhythm (slow and fast) buried within the theta cycles (**Figures 7A,B**, respectively). The theta frequencies driven by ZAP protocol were separated by 6 sections (0–2 Hz, 2–4 Hz…and so on, up to 10–12 Hz). The theta-gamma coupling properties were evaluated from each section in Non-TG and TG animals (**Figures 7C,D**). Peak MI was the maximum value of modulation indexes from all six-frequency distribution. The fast gamma (FG) and slow gamma (SG) peak modulation index from Non-TG group were significantly higher than that from the TG group (FG Non-TG: 15.03 ± 3.03; FG TG: 2.83 ± 1.05; SG Non-TG: 13.44 ± 1.91; SG TG: 1.82 ± 0.26; *n* = 5 in each group, two tailed *t*-test, *p* < 0.01, **Figure 7E**). The normalized values of theta power generated by the ZAP protocol were significantly higher in Non-TG group than TG group at (2–4 Hz, 4–6 Hz, 6–8 Hz), suggesting that the resonance properties in the hippocampal network oscillatory circuitry were greatly altered in the J20 group (**Figure 7F**).

### BACE1 Inhibitor (AZD3839) Rescued the Abnormal Phenotype Observed in J20 Mice

**FIGURE 8:**
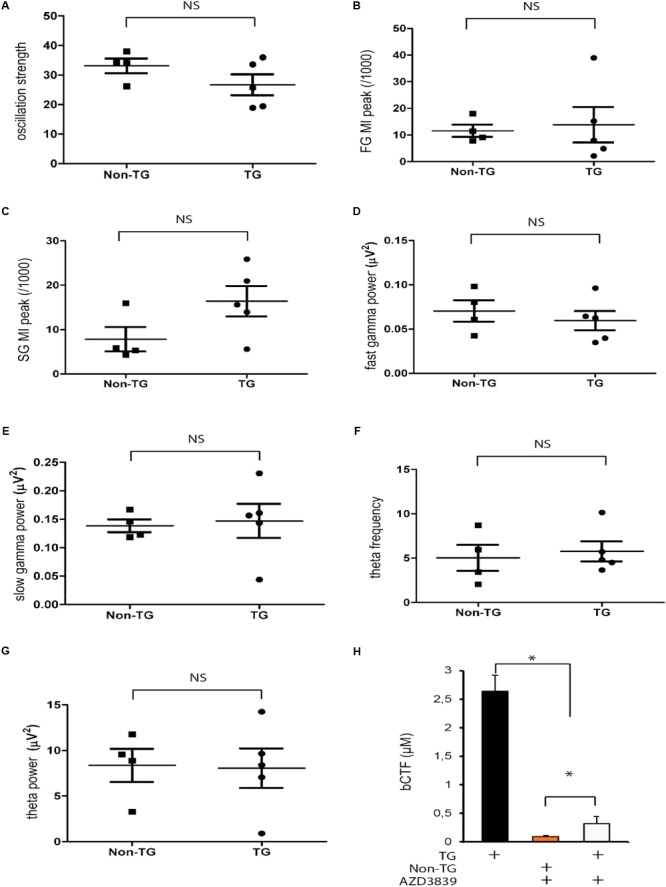
AZD3839 prevents b-CTF accumulation and rescues the abnormal phenotype in J20 mice. In J20-TG mice IP administrated AZD3839 rescues the theta strength alterations **(A)**. Theta-gamma CFC at slow (25–45 Hz) and fast (150–250 Hz) gamma range is also rescued in J20-TG treated mice **(B–E)**. Treated J20-TG mice did not show detectable changes in either, theta frequency **(F)** or theta power **(G)**, NS: *p* > 0.05. AZD3839 reduced b-CTF accumulation as detected by Elisa essay **(H)**, ^∗^*p* < 0.005.

To explore a potential rescue effect, we used an Aβ-secretase inhibitor AZD3839 (see section “Materials and Methods”). The inhibitor targets the first step in Aβ and b-CTF formation by directly blocking the action of BACE1 (Jeppsson et al., 2012). Due to its effectiveness, AZD3839 is currently in Phase I (Jeppsson et al., 2012; Evin and Hince, 2013). The mice received either vehicle or AZD3839 as a dose of 200 μmol/kg per day for 15 days (see section “Materials and Methods”). At ending point (p30) the neural network activity was monitored in the mice. AZD3839 increased the oscillation strength values in J20 mice, but not in Non-TG group (**Figure 8A**). Indeed, after AZD3839 treatment there was no longer significant differences between Non-TG and TG mice (*p* > 0.05, **Figure 8A**). Consistent with the above data, inhibiting BACE1 action also recovered the FG and SG peak values (**Figures 8B,C**). Again, after AZD3839 significant differences between control and TG mice were no longer found (*p* > 0.05, **Figures 8B,C**). Following the same trend, AZD3839 prevented alterations in FG and SG power (**Figures 8D,E**). As reported above, there were no alterations in either theta frequency or theta power (**Figures 8F,G**). To further confirm our data, we measured the b-CTF levels. After AZD3839 treatment, the levels of b-CTF were significantly decreased in treated TG mice (**Figure 8H**). Indeed, no differences between TG and Non-TG controls were observed (**Figure 8H**, *p* > 0.05). Importantly, no detectable levels of Aβ were detected either with or without AZD3839 inhibitor (data not shown).

## Discussion

This study identifies fundamental alterations in hippocampal network activity that are present as early as 30 days of age in the J20-AD mouse model (**Figure 1**). Of note, by 5 months of age J20 model exhibited the first traces of altered network activity, mostly reflected in network hyperexcitability and epileptiform activity (Verret et al., 2012). Accordingly, around the same age, the J20 model displayed the firsts deficits in learning, memory performance, synaptic alterations and Aβ deposition (Karl et al., 2012). However, our data showed that before any sign of Aβ accumulation, network dysfunction occurred. Specifically, at 1 month of age, J20 mice displayed abnormal oscillatory rhythmic activity (reduced theta strength) when compared to the Non-TG family related group (**Figure 1**). Surprisingly, J20 mice did not display significant changes in either theta frequency or theta power (**Figure 1**). It is likely, however, that rhythmicity – or oscillation strength – represents a more sensitive measure of network dynamics, which may better reflect coherence of cell assemblies as it considers the variations of both frequency and power of theta oscillations. These results therefore indicate that intrahippocampal homeostatic mechanism have been challenged but alterations are at initial stages, since preceding any memory impairment detectable by current available methods. In other words, the early alterations in rhythmic activity in J20 mice could potentially reflect the prodromal stage of AD. Of note, it has been shown that synchronous activity of hippocampal CA1/subiculum is critical for learning and long term memory formation (consolidating episodic memory) (Ognjanovski et al., 2017). In people with elevated risk of developing AD, abnormal brain activity of networks during memory encoding has been found at the prodromal stage of AD (Yafeng et al., 2016). Therefore, the early detected changes in rhythmic activity reported here could represent a pivotal stage which leads to cognitive impairment in the latter development of AD.

### Theta-Gamma Oscillations Phase Coupling and Network Stability

Understanding how the brain processes external and internal information is key to unraveling how brain regions communicate. Oscillatory activity within and across brain regions have been proposed to exert this function (Bonnefond et al., 2017). Indeed, is has been suggested that cross-frequency phase-amplitude coupling serves as a neural mechanism for coordinated working memory storage and maintenance (Daume et al., 2017). Not surprisingly, alterations in theta-gamma CFC may translate into cognitive deficit. In this regard, our data show that young J20 mice intrinsically displayed significant reduction in theta-gamma CFC within the slow (25–45 Hz) and fast (150–250 Hz) frequency gamma range (**Figure 2**). These data point to key alterations in neural mechanism for memory storage and maintenance. In agreement to our previous findings in a similar model (Mahar et al., 2016), the J20 mice displayed significant changes in the magnitude of theta-gamma CFC (**Figure 2**). Importantly, the magnitude of theta-gamma CFC has been correlated with working memory in rats and humans (Canolty et al., 2006; Scheffer-Teixeira and Tort, 2016). Thus, higher cognitive functions depend critically on synchronized network activity in the gamma range (30–200 Hz), which results from activity of fast-spiking PV+ cells. Consistent with the above data, we did find a reduction in the excitability of PV+ cells from J20 mice that could partly account for the instability of network theta oscillations (**Figure 3**).

As previously mentioned, at this age our J20 mouse did not display any detectable signs of memory impairment. Therefore, we propose that electrophysiological alterations observed in the J20 mouse, which include alterations in theta rhythmicity and theta-gamma CFC, could account for the early changes observed during the prodromal stage of AD. Importantly, the patients that develop AD are characterized by a pre-clinical phase that takes decades before any detectable sign of cognitive deficit (Van Der Hiele et al., 2007; Moretti et al., 2010). We therefore propose that our detected alterations in hippocampal oscillatory activity could represent a reliable biomarker for early diagnosis and potential therapeutic target.

### b-CTF and Network Dysfunction

As previously reported by other groups, soluble Aβ has been related to network alterations in J20 mice at 5-months of age (Verret et al., 2012; Palop and Mucke, 2016). However, in this mouse model it is well known that soluble Aβ is not detected before 4-months of age (Verret et al., 2012). Accordingly, our own Aβ assessment in young J20 mice was negative (**Figure 4**). No detectable levels of Aβ were found around the hippocampal formation in 1-month old J20 mice (**Figure 4**). Aiming to find an alternative mechanism responsible for electrophysiological alterations in J20 mice, we explored the presence of Aβ precursors (Cavanagh et al., 2013). Aβ peptides are generated by sequential proteolysis of hAPP by β and γ secretase (Vassar et al., 2009). The β site hAPP-cleaving enzyme 1 (BACE1) is the most important neuronal β secretase for Aβ aggregation (Vassar et al., 2009). The BACE1 action is the first hAPP cleavage and generates a b-CTF peptide, which is the intracellular C-terminal fragment. The intracellular b-CTF has been reported to be present as early as 30 days in TgCRND8-AD mouse model (Cavanagh et al., 2013). Likewise, in young J20 mice of the same age, intracellular b-CTF was found to be distributed along the entire PC layer from CA1, CA2, subiculum, DG and the entorhinal cortex (**Figure 5**). Importantly, this elevated concentration was found preferentially in the deep PC layer (**Figure 5**). Importantly, the b-CTF was barely detected in coexistence with PV+ interneurons (**Figure 5**). While we did find a reduction in the excitability of PV+ cells from J20 mice that could partly account for the instability of network theta oscillations, there were no major changes in the synaptic input and output properties of these cells. A significant difference, however, was observed in the frequency of spontaneous excitatory post-synaptic currents (sEPSCs) recorded in PV+ cells from control and J20-TG mice. While this suggests that there could be a change in the intrinsic or network properties of PCs, the fact that PV+ cells appeared not to be severely affected by b-CTF could explain the lack of change in the amplitude and frequency of theta in our model. An interesting possibility is that the decreased glutamatergic activity is indicative of a reduced glutamate release probability, in spite of the unaltered I/O relationship, or of a decrease in excitatory inputs coming from regions other than CA1 (such as the entorhinal cortex). Despite this data, the J20 mice displayed a peak of significant increase in b-CTF at 30 days of age and this increase remained present for up to 4 months (**Figure 6**). Mechanistically, it is known that b-CTF can directly interact with the heterotrimeric G protein Gαo, therefore, chronically Gαo activating by hAPP overexpression could elicit Go-dependent neurotoxic responses (Copenhaver and Kogel, 2017). Additionally, it has been reported that Gαo signaling activation enhances intracellular Ca^2+^ concentration (Ramirez et al., 2016). Further supporting this data, endogenous APP-Go interactions were found disrupted in AD patients (Copenhaver and Kogel, 2017). In this scenario, our detected intracellular b-CTF in the pyramidal neurons of the hippocampus could therefore translate in Ca^2+^ dysregulation that is reflected in network alterations.

Altogether, our studies showed that principal cells from the hippocampus are challenged as early as 30 days by intracellular Aβ precursor b-CTF, which in important proportion, could account for the very early alterations observed in rhythmic hippocampal activity.

Aiming at restoring hippocampal circuit coupling, we took advantage of the recent finding that PV+ optogenetic activation directly control theta power and frequency (Amilhon et al., 2015). We optogenetically manipulate theta activity in the J20 mice and evaluated the effect in theta-gamma CFC. Our data showed that low CFC values in our J20 mice were not restored by directly manipulating either, theta amplitude or theta frequency (**Figure 7**). Of note, our experimental preparation is lacking one of the main inputs coming from entorhinal cortex. Therefore, we proposed that entorhinal cortex is key in modulating gamma rhythms in the hippocampus. In this regard, it has been suggested that fast gamma rhythms in the hippocampus are coupled to fast gamma rhythms in the medial entorhinal cortex and may promote transmission of sensory information during new memory encoding (Colgin, 2016). Additionally, it has been shown that medial entorhinal cortex is necessary for temporal organization of hippocampal neuronal activity (Schlesiger et al., 2015). In summary, aiming to restore cognitive functions and memory, stimulation protocols need to take into consideration all sources of hippocampal inputs. Concomitantly, and further supporting the pathogenic role of b-CTF, pharmacological blocking of b-CTF generation restored the oscillatory alterations in our J20-TG model (**Figure 8**).

Notably, J20 mice showed impairments in PV+ interneurons and b-CTF accumulation in principal cells from hippocampus. Although the exact relationship between these two phenomena needs to be further elucidated, non-mutually exclusive possibilities could explain their coexistence (Kopec et al., 2006; Ripoli et al., 2014; Lei et al., 2016). Reduced glutamatergic transmission could be compensatory mechanism against b-CTF accumulation in principal cells.

## Conclusion

Our findings suggest that early APP processing generates intracellular fragments that impairs hippocampal rhythms as an initial challenge during AD development, resulting in remodeling of inhibitory circuits.

## Author Contributions

SM-R, NG, FM, and SW designed the experiments. SM-R, NG, and FM performed the experiments. SM-R, NG, FM, and SW analyzed the data.

## Conflict of Interest Statement

The authors declare that the research was conducted in the absence of any commercial or financial relationships that could be construed as a potential conflict of interest.

## Footnotes

1 https://hlab.stanford.edu/wdetecta.php
